# Chloride Ion Transport in Concrete Subjected to Sustained Compressive Stress Under Different Dry-Wet Ratios

**DOI:** 10.3390/ma18184388

**Published:** 2025-09-19

**Authors:** Wenqi Ma, Renchi Zhang, Xiang Li, Xiaokang Cheng, Yongming Xiong

**Affiliations:** 1 School of Civil Engineering, Changde Vocational Technical College, Changde 415000, China; xiaoma229297@163.com (W.M.);; 2 China Construction Fifth Engineering Bureau, Changsha 410004, China; 3 School of Civil and Environmental Engineering, Changsha University of Science and Technology, Changsha 410114, China; 4 Elite Engineering School, Changsha University of Science and Technology, Changsha 410114, China

**Keywords:** concrete, chloride transport, dry-wet time ratio, sustained compressive stress, chloride concentration

## Abstract

Existing studies on chloride ion transport in concrete under compressive load had rarely incorporated the influence of the dry–wet time ratio, even though this ratio was a key factor affecting chloride penetration in coastal concrete structures subjected to periodic drying–wetting cycles. This study was therefore motivated to fill this gap and to provide more reliable theoretical support for the durability assessment of such engineering structures. A series of accelerated chloride ion penetration experiments was conducted on concrete under compressive load with different dry–wet time ratios. The effects of the dry–wet time ratio, compressive stress level, and exposure environment on chloride ion transport in concrete were analyzed. A chloride ion diffusion coefficient model that accounted for both the dry–wet time ratio and the compressive stress level was then established and validated. The results showed that the enhancing effect of the dry–wet time ratio on chloride ion transport became significant under relatively high compressive stress. When the dry–wet time ratio was 7:1, the convection zone depths of concrete specimens under no stress and compressive stress were both 5 mm. Moreover, when the compressive stress level was 0.5 times the compressive strength and the dry–wet time ratio was 7:1, the chloride concentration of the specimens increased by an average of 756.4% compared with that under natural immersion.

## 1. Introduction

Chloride ions were highly corrosive species that destroyed the passive film of reinforcing steel and were widely distributed in concrete structures near marine environments [1,2,3]. Through numerous experiments and engineering applications, scholars concluded that the degree of corrosion in concrete structures subjected to both external loads and chloride environments was higher than that observed when only the chloride environment was considered [4]. Chloride ions penetrated concrete through several mechanisms, among which diffusion was the most common [5]. Therefore, it was important to investigate chloride transport in concrete under load.

Existing research on chloride diffusion under external load was mainly experimental [6,7,8,9]. Cheng et al. [6] investigated the distribution of chloride ions in concrete subjected to sustained compressive load and obtained the relationship between the diffusion coefficient and the compressive stress level. Turgeon-Malette et al. [7] examined the chloride ion permeability of ultra-high-performance fiber-reinforced concrete under sustained bending by combining digital image correlation to quantify multiple microcracks with an adapted accelerated migration test on loaded beams. Yoo et al. [8] evaluated chloride diffusion in concrete containing a cold joint under compressive and tensile loading, with and without 40% GGBFS (Ground Granulated Blast Furnace Slag), using accelerated migration tests. Wan et al. [9] applied four compressive stress levels to analyze the effect of compressive stress on chloride ion transport. Their study showed that when the stress level exceeded 0.3 *f_c_* (where *f_c_* referred to the compressive strength of concrete), chloride ion transport accelerated.

Although these studies discussed chloride transport mechanisms in concrete subjected to compressive load and drying–wetting cycles, limited research focused on the influence of the dry–wet time ratio on chloride transport under compressive stress. Drying–wetting cycles significantly influenced chloride ingress in concrete, particularly in marine environments. These cycles involved alternating periods of wetting and drying. Repeated wetting allowed chloride ions to penetrate the concrete, whereas drying caused water evaporation, salt crystallization, and expansion, which generated internal stresses and microcracks. This process increased the permeability of concrete, facilitated deeper chloride penetration, and enhanced the risk of reinforcement corrosion [10,11,12]. Rong et al. [13] reviewed the development of chloride transport models and emphasized that the influence of the dry–wet time ratio could not be neglected when analyzing chloride transport in concrete under compressive load. Therefore, it was necessary to investigate the influence of the dry–wet time ratio on chloride transport in concrete subjected to compressive stress.

Chloride diffusion coefficient models were indispensable for theoretical research and numerical simulations. Wang et al. [14] established chloride diffusion coefficient models for concrete under compressive load at different exposure periods. Chen et al. [15] conducted experiments on chloride transport in concrete subjected to sustained load and proposed a stress-dependent diffusion coefficient model. Although these studies considered the influence of stress level when developing chloride diffusion coefficient models, they rarely incorporated the effect of the dry–wet time ratio. Therefore, it was essential to establish a chloride diffusion coefficient model that accounted for both the dry–wet time ratio and the compressive stress level.

The purpose of this study was to propose a chloride ion diffusion coefficient model that considered both the dry–wet time ratio and the compressive stress level. A series of accelerated chloride ion penetration experiments was conducted on concrete subjected to compressive load under different dry–wet time ratios. The effects of the dry–wet time ratio, the compressive stress level, and the exposure environment on chloride ion transport in concrete were analyzed. Finally, a chloride ion diffusion coefficient model that incorporated both the dry–wet time ratio and the compressive stress level was established and validated.

## 2. Experiment Design

### 2.1. Materials and Design

In this study, Portland cement (CEM I) was used as the binder, with a standard strength grade of 42.5 MPa, and Table 1 presented detailed information on its chemical compositions and physical properties. Among these properties, the particle size distribution of the cement conformed to the Fuller gradation curve, and its fineness (an index reflecting particle size distribution) was determined by the sieve residue on a 45 μm square-hole sieve, which was 6.5%. Granite was selected as the coarse aggregate, with a particle size range of 5–20 mm, an apparent density of 2480 kg/m^3^, and a crushing index of 5.4%. River sand was used as the fine aggregate, with an apparent density of 2534 kg/m^3^, a bulk density of 1539 kg/m^3^, and a fineness modulus of 2.4. A polycarboxylate superplasticizer (SP) with a water-reducing rate of 20% was incorporated to enhance the workability of the concrete.

The designed concrete strength grade was NC45 with a water-to-cement ratio of 0.4, and a batch of cubic specimens with dimensions of 100 mm^3^ was prepared. The concrete mix proportions were provided in Table 2. After casting, the specimens were demoulded after 24 h and then cured at 20 °C and 95% relative humidity for 28 days. Subsequently, three concrete specimens were prepared for compressive strength testing, and the measured compressive strengths are reported in Table 2.

### 2.2. Chlorine Salt Erosion Experiment

Coastal cities experienced dry conditions throughout the year, and the monthly dry–wet time ratio of their coastal concrete structures varied significantly [16]. Concrete structures subjected to uniaxial sustained compressive stress were typically bridge piers. Existing studies indicated that the dry–wet time ratio of concrete structures in coastal areas affected by stress generally ranged from 1:1 to 13:1 [9,17]. To simultaneously investigate the effects of the dry–wet time ratio (*dw*), the compressive stress level (*λ*), and the exposure environment on chloride ion transport in concrete, this study designed specimen groups as shown in Table 3, which included 12 drying–wetting cycle groups and 4 natural immersion groups. Each group of concrete consisted of cubic specimens with dimensions of 100 mm^3^, and three specimens were prepared for each group, resulting in a total of 48 specimens.

**Figure 1:**
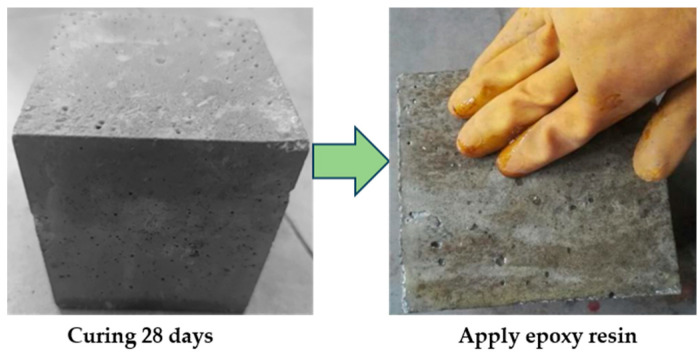
Preparation of concrete specimen.

**Figure 2:**
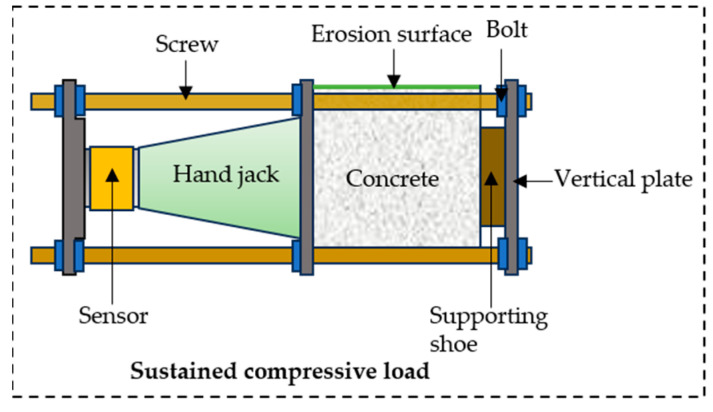
Apply compressive stress to concrete.

After the concrete specimens were cured, their surfaces were coated with epoxy resin, leaving only the top surface exposed to chloride penetration, as shown in Figure 1. Anti-corrosion treatment was applied to the components of the loading device, including the hand-operated jack, screw rods, sensors, and loading plates. Uniaxial loads corresponding to 0.2, 0.3, and 0.5 times the characteristic compressive strength of concrete (0.2, 0.3, and 0.5 *f_c_*) were subsequently applied to the specimens. Stress variations were monitored and recorded in real time by the sensors. Observations of stress changes were conducted at one-hour intervals until the applied load stabilized at the preset values without further fluctuations, as shown in Figure 2.

**Figure 3:**
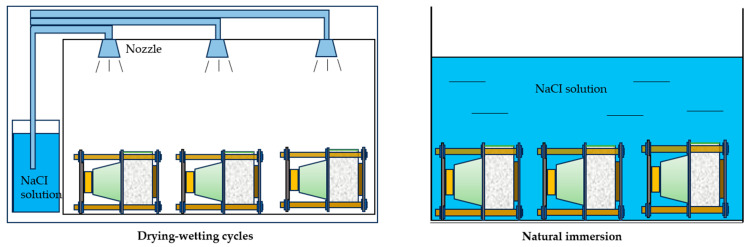
The concrete specimens with and without sustained load were put into a chloride environment.

The specimens were then divided into 12 drying–wetting cycle groups and 4 natural immersion groups, with three specimens included in each group to ensure the reliability of the test results. The drying–wetting cycle experiments were carried out in an artificial climate simulation chamber. For the loaded specimens, the upper surface was sprayed with a 5% NaCl solution, and three dry–wet time ratios (1:1, 3:1, and 7:1) were adopted. One complete drying–wetting cycle lasted for one day, with natural air drying used during the drying phase. The temperature in both the artificial climate simulation chamber and the natural immersion pool was maintained at 20 °C, with a relative humidity of 40% during the drying phase in the chamber. For the natural immersion group, a 5% NaCl solution was used as the immersion medium. The total duration of the experiment was 180 days, as shown in Figure 3. According to Al-Ameeri [18], in environments with low CO_2_ concentrations, the carbonation depth was shallow, and its influence on chloride diffusion was less pronounced. Therefore, carbonation effects were not considered in this study.

After the drying–wetting cycle and natural immersion experiments were completed, the corroded surfaces of the concrete specimens were sampled according to the procedure reported by Kosalla et al. [19]. To prepare concrete solutions for chloride concentration measurement, the protocol specified in ASTM C1218 [20] was followed. A drilling device was used to collect concrete samples from designated regions, ensuring representative sampling at different depths (e.g., at 2 mm intervals within the 0–20 mm range and 5 mm intervals within the 20–30 mm range). The collected samples were ground and sieved through a 75 μm sieve to achieve uniformity. For chloride ion extraction, a predetermined mass of powdered sample was mixed with deionized water at a fixed liquid-to-solid ratio (typically 10:1, using 5 g of powder and 50 g of water) and stirred continuously for 30–60 min until complete dissolution [21,22]. The chloride concentration of the test solution was then determined using an automatic potentiometric titrator.

## 3. Analysis of Experiment Results

### 3.1. Effect of the Dry-Wet Time Ratio

**Figure 4:**
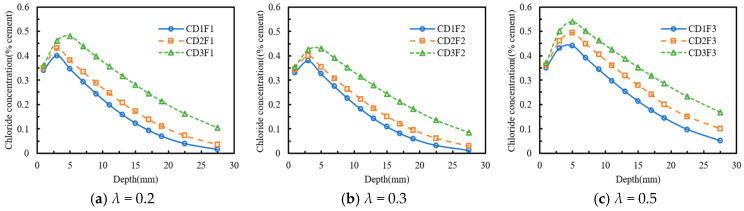
Effect of the dry-wet time ratio on chloride concentration distribution. (**a**) *λ* = 0.2; (**b**) *λ* = 0.3; (**c**) *λ* = 0.5.

Figure 4 illustrates the influence of the dry–wet time ratio on chloride diffusion in concrete subjected to sustained compressive stress. In Figure 4a, the effect of the dry–wet time ratio on chloride transport was evident, with the peak chloride concentrations of the CD1F1, CD2F1, and CD3F1 specimens measured as 0.40%, 0.43%, and 0.48%, respectively. The depth of the convection zone for both CD1F1 and CD2F1 specimens was 3 mm, whereas that of the CD3F1 specimen was 5 mm. The chloride concentrations of the CD2F1 and CD3F1 specimens increased by 27.45% and 74.52% on average, respectively, compared with that of the CD1F1 specimen.

In Figure 4b, when the compressive stress level was 0.3 *f_c_*, the differences in chloride concentration among the CD1F1, CD2F1, and CD3F1 specimens decreased. The possible explanation was that, at a compressive stress of 0.3 *f_c_*, the number of closed microcracks in the concrete specimens increased, thereby impeding chloride ion transport [17,23]. Although an increase in the dry–wet time ratio led to further chloride accumulation in the surface layer of the concrete, the densification of the concrete structure reduced the rate of chloride increase. The chloride concentrations of the CD2F2 and CD3F2 specimens increased by 25.83% and 72.35% on average, respectively, compared with that of the CD1F2 specimen.

As shown in Figure 4c, the chloride concentrations of the CD2F3 and CD3F3 specimens increased by 30.58% and 78.89% on average, respectively, compared with that of the CD1F3 specimen. This indicated that an increase in the compressive stress level promoted chloride ion diffusion. In summary, under the same compressive stress level, an increase in the dry–wet time ratio accelerated chloride ion transport. The enhancing effect of the dry–wet time ratio on chloride ion transport became significant under relatively high compressive stress.

### 3.2. Effect of the Compressive Stress Level

**Figure 5:**
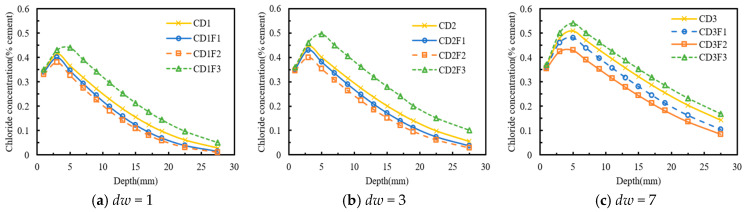
Effect of the compressive stress level on chloride concentration distribution. (**a**) *dw* = 1; (**b**) *dw* = 3; (**c**) *dw* = 7.

Figure 5 depicts the influence of compressive stress level on chloride diffusion in concrete subjected to sustained compressive stress. According to Figure 5a, when the compressive stress level increased from 0 to 0.5 *f_c_*, the peak chloride concentration of the CD1F3 specimen increased by 6.02% compared to that of the CD1 specimen, while the peak chloride concentrations of the CD1F1 and CD1F2 specimens decreased by 3.61% and 8.43%, respectively, compared to that of the CD1 specimen. In addition, the chloride concentration of the CD1F2 specimen was the lowest. The chloride concentrations of the CD1F3, CD1, and CD1F1 specimens increased by 82.82%, 32.81%, and 12.53% on average, respectively, compared to that of the CD1F2 specimen. In Figure 5b, with a dry-wet time ratio of 3:1, the influence of the compressive stress level on chloride concentration was greater than that in Figure 5a. The chloride concentrations of the CD2F3, CD2, and CD2F1 specimens increased by 85.83%, 35.8%, and 14.56% on average, respectively, compared to that of the CD2F2 specimen. Similarly, for Figure 5c, the chloride concentrations of the CD3F3, CD3, and CD3F1 specimens increased by 56.62%, 27.72%, and 11.52% on average, respectively, compared to that of the CD3F2 specimen. Moreover, when the dry-wet time ratio was 7:1, the convection zone depths of the CD3, CD3F1, CD3F2, and CD3F3 specimens all reached 5 mm.

To conclude, at the same dry-wet time ratio, chloride diffusion was relatively slow when the compressive stress level was 0.3 *f_c_*. As the dry-wet time ratio increased, the trend of chloride concentration first decreasing and then increasing with the compressive stress level became more obvious. When the dry-wet time ratio was 7:1, the convection zone depths of concrete specimens under no stress and compressive stress were 5 mm.

### 3.3. Effect of the Exposure Environment

**Figure 6:**
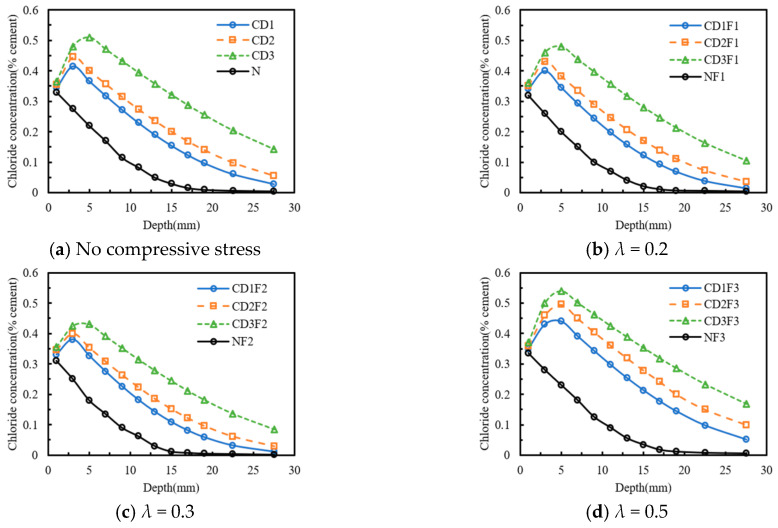
Effect of the exposure environment on chloride concentration distribution. (**a**) No compressive stress; (**b**) *λ* = 0.2; (**c**) *λ* = 0.3; (**d**) *λ* = 0.5.

Figure 6 shows the effect of the exposure environment on chloride diffusion in concrete subjected to sustained compressive stress. As illustrated in Figure 6a, the chloride concentrations of the CD1, CD2, and CD3 specimens were higher than those of the N specimen. Specifically, the chloride concentrations of the CD1, CD2, and CD3 specimens were on average 2.67, 3.41, and 5.50 times greater, respectively, than that of the N specimen. In Figure 6b–d, the chloride concentrations of specimens subjected to drying–wetting cycles were significantly higher than those of specimens under natural immersion. When the dry–wet time ratio was 7:1 and the compressive stress level was 0.5 *f_c_*, the chloride concentration of the CD3F3 specimen increased by 756.4% on average compared with that of the NF3 specimen. In conclusion, the effect of the drying–wetting cycling environment on the distribution of chloride concentration was more pronounced than that of the natural immersion environment.

## 4. Chloride Diffusion Coefficient Model

### 4.1. Establishment of Model

The chloride transport process before reaching the convection zone depth is relatively complex and influenced by moisture transfer, with the underlying mechanism being intricate [24]. The distribution model of free chloride concentration in surface concrete is shown in Equation (1) [25].

(1) $$C(x,t)=C_{s,\Delta x}\left[1-erf\left(\left(x-\Delta x\right)\cdot {\left(2\sqrt{\frac{D_{d,f}}{1-m}{\left(\frac{t_{0}}{t+t_{0}}\right)}^{m}\cdot t}\right)}^{-1}\right)\right]$$

where *C*(*x*,*t*) (%) is the free chloride concentration at a distance *x* (mm) (*x* > Δ*x*) from the concrete surface at time *t*; Δ*x* (mm) is the depth of the convection zone, determined experimentally; *C_s,_*_Δ*x*_ (%) is the peak chloride concentration; *t*_0_ (s) is the curing period; *t* (s) is the experiment period; *m* is the time decay coefficient, typically taken as 0.37 [24]; and *D_d,f_* (m^2^/s) is the equivalent apparent chloride diffusion coefficient under the combined action of the dry-wet time ratio and sustained compressive stress. In reference [26], the chloride diffusion coefficient considering the action of multiple factors is constructed as the product of the influence functions of several parameters and the undetermined chloride diffusion coefficient. This approach was also adopted in this study, which is expressed by Equation (2).

(2) $$D_{d,f}=D_{0}\cdot f(dw)\cdot f(\lambda )$$

where *D*_0_ (m^2^/s) is the apparent chloride diffusion coefficient to be determined; *f*(*dw*) is the influence coefficient considering the effect of the dry-wet time ratio; *f*(*λ*) is the influence coefficient considering the effect of the compressive stress level. The opening studies have modelled the chloride diffusion coefficient in saturated concrete subjected to sustained compressive stress, which is not considered in this study for the time being.

The convective zone depths of some specimens could be obtained from Figure 3, which are presented in Table 4.

The peak chloride concentration of concrete specimens was described in Table 5.

Furthermore, in Equation (1), the values of parameters *t*_0_ and *t* are determined as 2,419,200 s and 15,552,000 s, respectively. The data from Table 4 and Table 5 are substituted into Equation (1) to calculate the chloride diffusion coefficients of all concrete specimens. The calculated results were presented in Table 6.

The MATLAB 2016b toolbox was used for fitting analysis. Through the fitting process, it is found that the following equation could describe the chloride diffusion coefficients under different dry-wet time ratios.

*D_d,f_* = 1.11 × 10^−11^ + 3.5 × 10^−12^ *dw* R^2^ > 95% (3)

Further sorting out Equation (3) can obtain Equation (4).

*D_d,f_* = 1.11 × 10^−11^ (1 + 0.315·*dw*) R^2^ > 95% (4)

The value 1.11 × 10^−11^ m^2^/s in Equation (4) is used as the reference value for *D*_0_. Based on the convective zone depths and peak chloride concentrations of other specimens shown in Figure 3, the chloride diffusion coefficients are calculated. The MATLAB toolbox is employed for the fitting analysis. The results show that the fitting performance is better when a quadratic function is adopted to describe the influence of the compressive stress level, which is expressed in Equation (5).

*D_d,f_* = *D*_0_·[1 + 0.31·*dw*]·[1 − 0.74·*λ* − 5.17·*λ*^2^ + 14.33·*λ*^3^] R^2^ > 95% (5)

The chloride concentration in the diffusion zone, considering the dry-wet time ratio and the sustained compressive stress level, can be obtained by Equation (5).

### 4.2. Verification of Model

To verify the accuracy of the chloride diffusion coefficient model proposed in this study, experimental data from Wang et al. [14] and Cao et al. [27] were utilized for validation. Additionally, a comparative validation was conducted by comparing the proposed model with the model developed by Chen et al. [28]. In the experiment conducted by Wang et al. [14], the dry–wet time ratio was 1:1, the sustained compressive stress levels were 0.2 and 0.7, and the exposure period was 112 days. In the experiment by Cao et al. [27], the dry–wet time ratios were 1:1 and 11:1, and the experimental period was 60 days. Based on the experimental results reported by Wang et al. [14] and Cao et al. [27], the depths of the convective zone and the chloride concentration peaks were determined. In the present study, calculations were performed using the chloride concentration peak as the input parameter.

**Figure 7:**
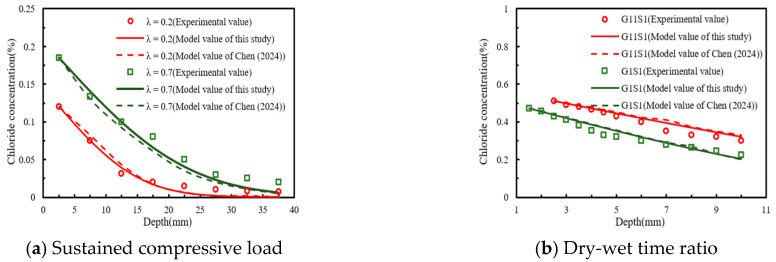
Comparative validation of this study with Chen (2024) [28] of the chloride diffusion model (**a**) Sustained compressive load; (**b**) Dry-wet time ratio.

Figure 7 depicts a comparative analysis of experimental and model values from the open literature. As shown in Figure 7a, the experimental and modelled values of chloride concentration were in closer agreement when the diffusion depth was within 12.5 mm. In contrast, the values predicted by the model proposed by Chen et al. [28] exhibited greater deviations from the experimental results. As the diffusion depth increased, the discrepancy between the experimental and model values also increased. The possible explanation was that, as the diffusion depth increased, the chlorides bound to the interior of the concrete matrix, which slowed the diffusion rate [6]. In Figure 7b, the error between the experimental chloride concentration values and the simulated values was small, and the experimental data were evenly distributed on both sides of the model-predicted values.

To further verify the applicability of the chloride diffusion coefficient model proposed in this study, the Mean Absolute Percentage Error (MAPE) was employed for evaluation. The relevant error results were presented in Table 7 and Table 8. From these data, it was observed that the MAPE values of Chen et al.’s model were larger, indicating higher prediction errors. Specifically, as shown in Table 5, the maximum error of the model proposed in this study occurred in the case of G11S1, with a corresponding MAPE value of 12.34%. In contrast, the MAPE value of Chen et al.’s model for the same case was 15.65%. According to the research of reference [29], when the MAPE value between experimental and model results was less than 15%, the model could be considered applicable. Therefore, the chloride diffusion coefficient model that incorporated both the dry–wet time ratio and sustained compressive stress proposed in this study successfully described the distribution law of chloride concentration in concrete subjected to compressive load under different dry–wet time ratios.

## 5. Conclusions

A series of accelerated chloride ion experiments was carried out on concrete subjected to compressive load under different dry–wet time ratios. The effects of the dry–wet time ratio, compressive stress level, and exposure environment on chloride ion transport in concrete were analyzed. Finally, a chloride ion diffusion coefficient model that considered both the dry–wet time ratio and compressive stress level was established and validated. The following main conclusions were drawn:

1. The enhancing effect of the dry–wet time ratio on chloride ion transport became significant under relatively high compressive stress. When the dry–wet time ratio was 7:1, the convection zone depths of concrete specimens under no stress and compressive stress were both 5 mm.
2. The effect of the drying–wetting cycling environment on the distribution of chloride concentration was more pronounced than that of the natural immersion environment.
3. When the compressive stress level was 0.5 *f_c_* and the dry–wet time ratio was 7:1, the chloride concentration of the specimens increased by 756.4% on average compared with that under natural immersion.
4. The Mean Absolute Percentage Error (MAPE) between the experimental results and the model predictions proposed in this study was less than 15%, and the accuracy of the proposed model was higher than that of existing models. Therefore, the chloride diffusion coefficient model considering both the dry–wet time ratio and sustained compressive stress was verified to be applicable.

This paper involved a limited number of working conditions and experimental data. In addition, the experimental results might have had a certain degree of discreteness. Therefore, for practical engineering, specific analyses should be conducted based on the conclusions of this paper in light of specific circumstances.

## Figures and Tables

**Table 1:** Chemical compositions and physical properties of cement.

	SiO_2_	Al_2_O_3_	Fe_2_O_3_	CaO	MgO	SO_3_	TiO_2_	K_2_O	Loss on Ignition
Chemical composition (%)	21.09	6.37	4.57	60.48	1.43	1.89	0.21	0.45	2.57

**Table 2:** Mix ratio and related parameters of concrete.

**NC45**	**Unit (kg/m^3^)**					***f_c_* (MPa)**
	**Water**	**Cement**	**Natural Fine Aggregate**	**Natural Coarse Aggregate**	**SP**	47.5
	180	450	550	1170	3	

**Table 3:** Experiment specimen number.

Specimens	Expose the Environment	Dry-Wet Time Ratio	Sustained Compressive Load (*f_c_*)
CD1	Drying-wetting cycles	1:1	-
CD2		3:1	-
CD3		7:1	-
CD1F1		1:1	0.2
CD1F2		1:1	0.3
CD1F3		1:1	0.5
CD2F1		3:1	0.2
CD2F2		3:1	0.3
CD2F3		3:1	0.5
CD3F1		7:1	0.2
CD3F2		7:1	0.3
CD3F3		7:1	0.5
N	Natural immersion	-	-
NF1		-	0.2
NF2		-	0.3
NF3		-	0.5

**Table 4:** Depth of convection zone in concrete specimen.

	CD1	CD2	CD3
Δ*x* (mm)	3	3	5

**Table 5:** The peak chloride concentration of concrete specimens.

	CD1	CD2	CD3
*C_s,_*_Δ_*_x_* (%)	0.415	0.446	0.511

**Table 6:** The value of *D_d,f_*.

	CD1	CD2	CD3
*D_d,f_* (m^2^/s)	1.462 × 10^−11^	2.17 × 10^−11^	3.57 × 10^−11^

**Table 7:** MAPE under different sustained compressive load.

Type	*λ* = 0.2 (Model of This Study)	*λ* = 0.2 (Model of Chen (2024) [28])	*λ* = 0.7 (Model of This Study)	*λ* = 0.7 (Model of Chen (2024) [28])
MAPE (%)	8.67	9.56	10.56	12.78

**Table 8:** MAPE under dry-wet time ratio.

Type	G11S1 (Model of This Study)	G11S1 (Model of Chen (2024) [28])	G1S1 (Model of This study)	G1S1 (Model of Chen (2024) [28])
MAPE (%)	12.34	15.65	9.67	10.45

## Data Availability

The original contributions presented in this study are included in the article. Further inquiries can be directed to the corresponding authors.
